# Measurement of vertebral rotation using a three-dimensional ultrasound image

**DOI:** 10.1186/1748-7161-10-S1-O40

**Published:** 2015-01-19

**Authors:** Quang N Vo, Edmond H M Lou, Lawrence H Le

**Affiliations:** 1 University of Alberta, Alberta, Canada; 2 Ho Chi Minh City University of Technology, Ho Chi Minh City, Vietnam

## Objective

Vertebral rotation (VR) is an important parameter to evaluate the severity of scoliosis and predict the progression. However, using two-dimensional radiographs to measure VR may under estimate its true value. This study investigated a new three-dimensional (3D) non-ionizing method to measure VR based on either laminae or transverse processes.

## Materials and methods

Three cadaveric vertebrae T7, L1, and L3 were scanned with a 3D medical ultrasound system. The rotation angles of the cadaveric vertebra were recorded during the experiment. Nine sets of ultrasound data, from 0 to 40° with 5° increments, were recorded from each vertebra (27 sets in total). An in-house program was used to reconstruct the 3D vertebra images. The rotation of each reconstructed vertebra was determined by the angle between the line going through either the centres of laminae (L-L) or the centres of transverse processes (TP-TP) and a reference vertical plane. This reference plane was defned from the position sensor parallel to the surface of the transducer. Three raters, who were blinded with the rotation information, used the images to measure the rotation in 3 sessions. In each session, the raters used the mouse pointer to select L-L or TP-TP according to their knowledge of vertebral anatomy. The program received the 3D coordinates of these points and calculated the VR.

Intra-class correlation coefficients (ICCs) (two-way random and absolute agreement) were used to calculate the intra- and inter-reliability. The mean absolute difference (MAD±SD) and the range of difference (RD) between the true values and the average measurements of each rater were also computed to evaluate the accuracy of methods.

## Results

When rotation was greater than 30° for both L1 and L3, all raters found it difficult to determine one of the lamina areas because it could not be displayed due to ultrasound blocking. Therefore, the corresponding measurements were excluded. The intra-reliability (L-L, TP-TP) for the three raters were (0.987, 0.991), (0.989, 0.998) and (0.997, 1.000), respectively; meanwhile, the inter-reliability were 0.991 for (L-L) and 0.992 for (TP-TP). All ICC values were greater than 0.98 indicating both methods were highly reliable. The MAD±SD values (L-L, TP-TP) for the three raters were (1.5±0.3°, 1.2±0.2°), (1.6±0.3°,1.3±0.3°), and (1.7±0.5°, 0.9±0.2°), respectively. The RD (L-L, TP-TP) were (0-4.5°, 0-3.5°), (0-5.1°, 0-4.3°), and (0-5.1°, 0-2.8°) for the three raters, respectively.

## Conclusions

The results demonstrated that L-L and TP-TP could be used interchangeably to measure VR from the 3D ultrasound images.

